# DUAL ROLES OF FITNESS AND FATIGABILITY IN THE LIFE-SPACE MOBILITY OF OLDER ADULTS

**DOI:** 10.1093/geroni/igac059.1457

**Published:** 2022-12-20

**Authors:** Kyle Moored, Yujia (Susanna) Qiao, Andrea Rosso, Frederico Toledo, Steven R Cummings, Bret Goodpaster, Stephen Kritchevsky, Nancy W Glynn

**Affiliations:** Johns Hopkins Bloomberg School of Public Health, Baltimore, Maryland, United States; University of Pittsburgh, Schoold of Public Health, Pittsburgh, Pennsylvania, United States; University of Pittsburgh, Pittsburgh, Pennsylvania, United States; University of Pittsburgh, Pittsburgh, Pennsylvania, United States; San Francisco Coordinating Center, San Francisco, California, United States; AdventHealth, Orlando, Florida, United States; Wake Forest School of Medicine, Winston-Salem, North Carolina, United States; School of Public Health, University of Pittsburgh, Pittsburgh, Pennsylvania, United States

## Abstract

Objective fitness and perceived fatigability may interact to limit mobility within the larger environment (life-space mobility). We assessed this cross-sectionally in SOMMA (N=371, mean age=76.4±5.0). Life Space Assessment scores (range: 0-120, 5-point difference=clinically relevant, e.g., going outside additional 1-3 days/week) incorporated level, frequency, and assistance used for life-space mobility (Mean=82.7±18.8). Fitness was measured as VO2peak (Mean=19.5±4.2 mL/kg/min) from a symptom-limited treadmill test. Higher fatigability was defined as a Borg Rating of Perceived Exertion (RPE, range: 6-20) ≥10 after a five-minute steady-state treadmill test. Each 1-SD higher VO2peak was associated with a 2.2-point higher life-space score (95% CI: 0.25-4.07, p=.027). After adjustment for demographic, lifestyle, and health confounders, the association between fitness and life-space mobility was significant only for those with higher fatigability (RPE≥10: B=5.70, 95% CI: 0.79-10.60, p-interaction=.008). Older adults with both lower fitness (objective capacity) and higher fatigability (perceived capacity) may be at greatest risk of reduced real-world mobility.

